# Early neurophysiological biomarkers and spinal cord pathology in inherited prion disease

**DOI:** 10.1093/brain/awy358

**Published:** 2019-01-28

**Authors:** Peter Rudge, Zane Jaunmuktane, Harpreet Hyare, Matthew Ellis, Martin Koltzenburg, John Collinge, Sebastian Brandner, Simon Mead

**Affiliations:** 1 National Prion Clinic, National Hospital for Neurology and Neurosurgery, University College London Hospitals NHS Foundation Trust (UCLH), London, UK; 2 MRC Prion Unit at UCL, Institute of Prion Diseases, 33 Cleveland St. London, UK; 3 Division of Neuropathology, National Hospital for Neurology and Neurosurgery, University College London NHS Foundation Trust, Queen Square, London, UK; 4 University College London NHS Foundation Trust, Queen Square, London, UK; 5 Department of Neurodegenerative Disease, UCL Institute of Neurology, Queen Square, London, UK

**Keywords:** P102L, GSS, sensory symptoms, spinal cord

## Abstract

A common presentation of inherited prion disease is Gerstmann-Sträussler-Scheinker syndrome, typically presenting with gait ataxia and painful dysaesthesiae in the legs evolving over 2–5 years. The most frequent molecular genetic diagnosis is a P102L mutation of the prion protein gene (*PRNP*). There is no explanation for why this clinical syndrome is so distinct from Creutzfeldt-Jakob disease, and biomarkers of the early stages of disease have not been developed. Here we aimed, first, at determining if quantitative neurophysiological assessments could predict clinical diagnosis or disability and monitor progression and, second, to determine the neuropathological basis of the initial clinical and neurophysiological findings. We investigated subjects known to carry the P102L mutation in the longitudinal observational UK National Prion Monitoring Cohort study, with serial assessments of clinical features, peripheral nerve conduction, H and F components, threshold tracking and histamine flare and itch response and neuropathological examination in some of those who died. Twenty-three subjects were studied over a period of up to 12 years, including 65 neurophysiological assessments at the same department. Six were symptomatic throughout and six became symptomatic during the study. Neurophysiological abnormalities were restricted to the lower limbs. In symptomatic patients around the time of, or shortly after, symptom onset the H-reflex was lost. Lower limb thermal thresholds were at floor/ceiling in some at presentation, in others thresholds progressively deteriorated. Itch sensation to histamine injection was lost in most symptomatic patients. In six patients with initial assessments in the asymptomatic stage of the disease, a progressive deterioration in the ability to detect warm temperatures in the feet was observed prior to clinical diagnosis and the onset of disability. All of these six patients developed objective abnormalities of either warm or cold sensation prior to the onset of significant symptoms or clinical diagnosis. Autopsy examination in five patients (including two not followed clinically) showed prion protein in the substantia gelatinosa, spinothalamic tracts, posterior columns and nuclei and in the neuropil surrounding anterior horn cells. In conclusion, sensory symptoms and loss of reflexes in Gerstmann-Sträussler-Scheinker syndrome can be explained by neuropathological changes in the spinal cord. We conclude that the sensory symptoms and loss of lower limb reflexes in Gerstmann-Sträussler-Scheinker syndrome is due to pathology in the caudal spinal cord. Neuro-physiological measures become abnormal around the time of symptom onset, prior to diagnosis, and may be of value for improved early diagnosis and for recruitment and monitoring of progression in clinical trials.

## Teaser

A common presentation of the P102L prion protein mutation is Gerstmann-Straussler-Scheinker syndrome. Rudge *et al.* show that serial measurement of lower limb thermal thresholds predicts, within a relatively narrow time window, conversion to the symptomatic phase of the disease, and that synaptic deposition of PrP^Sc^ in the spinal cord underpins the pathology.

## Introduction

Prion diseases, or transmissible spongiform encephalopathies, are a group of fatal neurodegenerative disorders affecting humans and animals (Collinge, 2001). The central mechanism of prion diseases is the conversion of host encoded prion protein (PrP^C^) to a misfolded multimeric state (Prusiner, 1982). Human prion diseases occur in sporadic, acquired and inherited forms. The majority develop progressive neurodegeneration with cognitive impairment and other neurological deficits primarily, but not exclusively, due to CNS dysfunction. Sporadic and acquired prion diseases generally follow a rapid course lasting less than 2 years. In some, although not all, hereditary forms the duration of the disease is much longer ranging from 2 years, to more than 20 years.

Inherited prion diseases are caused by ∼40 mutations in the prion protein gene (*PRNP*) and are highly heterogeneous in clinical onset, manifestation and duration, often with considerable variation within a single family, including several examples of a child presenting before the development of disease in a parent (Webb *et al.*, 2008). Some heterogeneity can be explained by the mutation type and by genetic variation elsewhere in *PRNP* and some is correlated with biochemical properties of PrP^Sc ^(Parchi *et al.*, 1998; Kovacs *et al.*, 2002; Mead, 2006; Wadsworth *et al.*, 2006). In the UK, the P102L mutation is one of the most frequently encountered mutations and has been shown to be the cause of Gerstmann-Sträussler-Scheinker disease, a term that has been extended to other mutations (Hsiao *et al.*, 1989; Bugiani *et al.*, 2000). Patients usually present with ataxia, especially of gait, and lower limb sensory disturbance in mid-late adulthood. Later cognitive decline occurs together with multiple CNS symptoms culminating in an akinetic mute state after a duration of ∼4 years (Webb *et al.*, 2008). In this retrospective series (84 patients) with clinical evidence of weakness and muscle wasting, thought to be indicative of lower motor neuron dysfunction, occurred in 22% but nearly all had pyramidal signs; only one of eight studied neuro-physiologically had evidence of denervation and 2 of 10 had evidence of large fibre axonal neuropathy. The sole consistent neurophysiological finding was absence of the H reflex from tibial nerve stimulation in the four patients in whom it was sought. Interestingly, thermal threshold testing was abnormal in the lower limbs in the one patient tested. Further neurophysiological studies have been reported in seven patients (Salsano *et al.*, 2011). The only abnormality in this series was a loss of the H reflex in the lower limbs in the four who were tested reflecting the areflexia. Large fibre peripheral conduction in motor and sensory nerves and somatosensory evoked potentials were normal. Based on these neurophysiological studies, an imaging study (Arata *et al.*, 2006) and a single morphological case study (Yamada *et al.*, 1999), a caudal myelopathic process as an underlying cause of areflexia and sensory loss in patients with Gerstmann-Sträussler-Scheinker disease due to *PRNP *P102L mutation has been proposed. However, detailed confirmatory morphological studies in a case series, regarding the extent and distribution of the underlying pathology have not been done.

Here we report the prospective longitudinal clinical and neurophysiological findings in 23 patients carrying the P102L mutation, including the development of symptom onset, diagnosis and disability in six and autopsy in five. The patients are members of the large UK pedigrees which we have followed for over 20 years (Webb *et al.*, 2008). There were two objectives of this study. First, to determine if alterations in neurophysiological parameters predict the onset of clinical disease. The development of such measures is important since potential treatments, including those being produced for prion diseases, are likely to be more effective if given before extensive neuronal damage has occurred. In other inherited neurodegenerative diseases prediction of clinical onset has relied on family history (Alzheimer’s disease) or length of DNA repeat regions (Huntington’s disease). The situation is even more difficult in the inherited prion diseases because age at onset variation is high (onset for the P102L associated syndrome in the pedigree we studied ranges from ages 26 to 71, standard deviation ∼10 years), and criteria are not optimized for an early diagnosis. Current criteria for diagnosis of inherited prion diseases were designed for epidemiological purposes and require a 6-month history of an progressive neuropsychiatric syndrome (NCJDRSU, 2017). Inherited prion disease due to a P102L mutation is well suited to develop neurophysiological biomarkers as patients appear to present with lower limb areflexia and sensory disturbance before any significant neurological impairment occurs. Further, serial neuro-physiological parameters might be a valuable objective marker of disease progression during therapeutic trials. Our second objective was to correlate the clinical and neurophysiological findings with neuropathological abnormalities. In particular, we aimed at understanding the cause of the areflexia and sensory loss and how this could be matched with the anatomy of prion protein deposition and neurodegeneration in the peripheral and spinal tracts.

## Materials and methods

### Patient cohort and clinical assessment

The National Prion Monitoring Cohort Study (Cohort Study) was established in 2008 and is an observational longitudinal study of patients with, or at risk of, developing human prion disease (sporadic, acquired and inherited prion diseases) (Thompson *et al.*, 2013). All eligible patients referred to the National Prion Clinic (NPC) are invited to take part in the Cohort Study, which involves review by a NPC consultant neurologist or clinical research fellow and collection of systematic clinical data including physical examination findings, blood tests, neuropsychology, neurophysiology and MRI at regular intervals depending on the type of prion disease. In the case of P102L inherited prion disease, the interval would typically be 12 months in asymptomatic mutation carriers and 6 months in symptomatic disease. Systematic history taking, rating scale analysis and review of clinical letters defined three milestones: (i) onset of any symptom that subsequently developed into part of the Gerstmann-Sträussler-Scheinker syndrome; (ii) onset of disability, defined as an MRC Prion Disease Rating Scale score <20; and (iii) the time at which the NPC clinician confirmed the diagnosis of Gerstmann-Sträussler-Scheinker with patient and family. The definition of (i) is complicated because gene mutation carriers have frequent, non-progressive psychological/psychiatric comorbidities long before clinical onset; however, psychiatric symptoms can be a presenting feature of Gerstmann-Sträussler-Scheinker (Thompson *et al.*, 2014).

### Molecular genetics

From the Cohort Study of 31 patients at risk of carrying the mutation, 23 were sequenced for *PRNP* (Wadsworth *et al.*, 2017). Informed consent for the analysis was obtained in all cases. *PRNP* includes a common nucleotide polymorphism in European populations which encodes for either methionine or valine at codon 129; the genotype at this position was determined as part of sequencing the *PRNP* open reading frame.

### MRI analysis

MRI studies performed at either 1.5 or 3 T were reviewed. The images were reviewed by a consultant neuro-radiologist (H.H.) and a consultant neurologist (P.R.), both with 9 years’ experience in prion disease imaging, and agreement was achieved via consensus. Signal abnormality was assessed in the caudate, putamen and thalamus on T_2_-weighted, FLAIR and/or diffusion-weighted imaging (DWI) sequences. Cortical signal abnormality was assessed on DWI sequences where available in the following areas: frontal, parietal, temporal, occipital, cingulate, insula, hippocampus and cerebellum. Finally the T_1_-weighted images were reviewed for either focal or generalized supra-tentorial and/or cerebellar atrophy.

### Neurophysiological studies

Standard nerve conduction studies (Preston, 2013) were performed on a Nicolet Viking Select (Natus) with temperatures >30° and 32° in upper and lower limbs respectively. Motor nerve conduction studies and F-waves were recorded as baseline to negative peak using the belly tendon montage and a terminal conduction distance of 80 mm. Sensory responses were recorded antidromically from the sural (retromalleolarly) and the superficial peroneal nerve at a conduction distance of 120 mm and orthodromiclly between the base of digits 2, 3 and 5 to the median or ulnar nerve at the wrist. For recording of the H-responses the patient was prone or in the lateral position with the knee flexed at 30° and asked to clench teeth and fists. M- and H-responses were recorded from the calf muscles following graded electrical stimulation at the midpoint of the back of the knee using a stimulus duration of 1 ms. The magnitude of the reflex response was expressed as the ratio H/M amplitude measured peak to peak.

Thermal thresholds were recorded using a TSA-II (Medoc, Ltd.) and a 30 × 30 mm thermode placed on the lateral dorsum of the foot or hand. The tests were performed using the method of limits with a baseline of 32°C, a change of temperature of 1°C, and upper and lower limits of 55°C and 0°C, respectively. Thresholds were defined as the average of three trials for cold (CDT) and warm (WDT) detection thresholds. The sequence of testing was CDT, WDT in hands and feet (Rolke *et al.*, 2006).

Itch and neurogenic flare were evoked by the intracutaneous injection of 10 μl of 100 μg histamine chloride into the anterior aspect of the lower leg. The magnitude of the maximal itch sensation was scored on a 0–10 Likert scale. The flare was studied in a 10 × 10 cm area (1 × 1 mm pixel, scan duration ∼5 min) with a Laser Doppler Imager (moorLDI, Millwey). The area of the flare was expressed as the area of pixels exceeding the mean + 3 (rather than 2 as described by Bickel *et al.*, 2002) standard deviations of the baseline.

### Neuropathological examination

Neuropathological studies were carried out on 3 of 11 symptomatic patients and on two additional P102L Gerstmann-Sträussler-Scheinker patients not included in the neurophysiological study. The brains, including the lower brainstem were removed in all five, the spinal cord of variable length in three and representative peripheral nerves in two patients. The sampled nerves were fixed in 10% buffered formalin for 1 week and the brainstem together with the spinal cords continued to be fixed for another 2 weeks. Then brains and spinal cords were sliced in coronal and transverse sections, respectively. Paraffin wax sections of 4-µm thickness were prepared and stained with haematoxylin and eosin and 14-µm thick sections were stained with Luxol fast blue/cresyl violet. Immunostaining for abnormal prion protein was carried out on 4-µm thick sections with anti-PrP ICSM35 (1:1000 of 100 μg/ml solution, D-Gen Ltd) and 12F10 (1:200 Cayman Chemical). ICSM35 recognizes residues 93–102, and is known not to bind to P102L mutant PrP, but does recognize PrP derived from the wild-type allele (Wadsworth *et al.*, 2006). 12F10 labels residues 142–160 of both wild-type human PrP and P102L PrP. To detect abnormal PrP deposits, deparaffinized sections were treated with 98% formic acid for 5 min, placed on an automated Ventana Discovery XT staining machine, and treated with Cell Conditioning Solution Plus and Protease 3, incubated in Superblock for 10 min, then exposed to the primary antibody followed by biotinylated anti-mouse IgG secondary antibody and visualised using the DAB Map Detection Kit (Ventana Medical System). Sections were counterstained with haematoxylin and coverslipped (LEICA CV5030 coverslipper). All stainings were carried out with appropriate controls.

### Evaluation of the abnormal prion protein deposits

In each case where tissue was available the pattern of the abnormal prion protein deposits (filamentous, granular or plaque-like) and the regional distribution of the prion pathology were assessed with particular emphasis on the following areas: spinal grey matter, spinothalamic pathway, posterior column-medial lemniscus pathway (PCML), anterior and posterior nerve roots, nerve roots in the cauda equina, peripheral nerves.

### Image capturing and analysis

For image capturing all histological slides were digitalized on a LEICA SCN400 scanner (LEICA UK) at ×40 magnification and 65% image compression setting. For assessment of the density of abnormal prion protein deposits on histological slides from the brainstem medulla at the level of gracile and cuneate nuclei digital image analysis was conducted using Definiens Tissue Studio 3.6 (Definiens). Regions of interest—gracile and cuneate nuclei—were selected manually in each case. Tissue Studio composer actions were used to automatically identify regions of interest. In each case the size of the region of interest and the intensity of the prion labelling was calculated and further compared between both nuclei.

### Ethics approval

Research ethics approval was obtained from Scotland MREC A. Informed consent to use the tissue for research was obtained in all instances.

### Data availability

The data that support the findings of this study are available from the corresponding author upon reasonable request.

## Results

Thirty-one patients with or at risk of inheriting the P102L mutation were recruited; 23 patients (16 females) with the mutation were studied neuro-physiologically. One gene mutation positive patient had a diabetic neuropathy potentially confounding the neurophysiological assessment and eight at risk of having the mutation were not genotyped. Sixteen patients were asymptomatic and six symptomatic when first seen. Six patients became symptomatic during follow up. At the start of the study the mean age of the symptomatic patients was 49.3 years (range 26–61) and the asymptomatic patients was 41.6 years (range 28–56). The maximum number of neurophysiological studies in any one patient was five over a period of up to 10 years.

### Genetic results

Thirteen patients with the mutation were codon 129 methionine homozygous, eight of whom were symptomatic at some stage during the study; eight were heterozygotes, three of whom were symptomatic at some time during the study; and in one patient the polymorphism at codon 129 is unknown.

### Clinical assessment

#### Patients symptomatic on presentation

The presenting symptoms in the symptomatic patients were predominantly ataxia of gait and pain or discomfort in the lower limbs. Myoclonus, cognitive decline and weakness were less prominent except in the youngest patient who presented with severe cognitive decline. At subsequent follow-up there was progression of pain or itching in legs, ataxia, myoclonus and cognitive decline to varying degrees (Webb *et al.*, 2008).

The findings on clinical examination at the first visit in those who were initially symptomatic, 1–18 months after first developing symptoms, are summarized in Table 1. Upper limb examination was initially normal in all. The neurological signs progressively worsened during follow-up and new signs developed, including myoclonus, cognitive decline and weakness (Table 1). Late in the course of the disease abnormal neurological signs developed in the upper limbs with loss of the tendon reflexes and muscle wasting; at this stage detailed sensory assessment was not possible because of cognitive decline. Five patients have died at 35–56 (mean 46.6) months after developing symptoms.

**Table 1:** Summary of clinical findings at first and final examination in patients who were symptomatic when referred

Time	Weakness	Myoclonus	Absent reflex	Extensor plantar	Vibration loss	Thermal loss	Ataxia	Cognitive decline
First	1/5	2/6	5/6	3/6	3/6	4/5	6/6	3/6
Last	3/5	5/6	6/6	4/5	5/6	5/5	5/5	6/6

#### Patients who converted from asymptomatic to symptomatic

**Figure 1:**
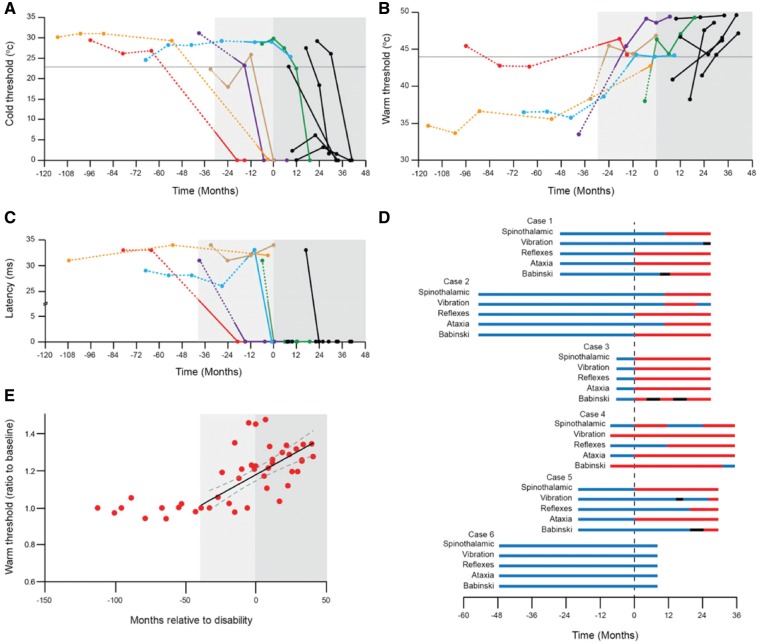
**Neurophysiological and clinical findings in patients who became symptomatic.** Serial measurement of cold threshold (**A**), hot threshold (**B**) and H reflex latency (**C**). Time of significant symptom onset (MRC Scale score <20) is indicated by dark grey shading at time 0. Time of first symptom is indicated in individual patients by a change from dashed to solid line, and from the time of first symptom in any patient with light grey shading. Months prior to significant symptoms shown as negative values; months after significant symptoms shown as positive values. Black lines are serial measurements of five cases symptomatic when first seen. Horizontal grey lines in **A** and **B** are 95% confidence measurement for thermal thresholds. Coloured lines are serial measurements in patients who converted during study: Case 1 red, Case 2 blue, Case 3 green, Case 4 brown, Case 5 purple, Case 6 orange. (**D**) Serial clinical findings prior to symptomatic conversion (negative values) and after conversion (positive values). Conversion indicated by broken line at time 0. Normal findings indicated in blue, abnormal findings in red, and no assessment in black. Spinothalamic refers to detection of cold or pain in lower limb, vibration refers to detection of vibration at the ankle, reflexes refers to ankle tendon reflex, ataxia refers to gait ataxia, and Babinski refers to plantar response. (**E**) Segmented linear model of thermal thresholds assuming no change 30 months prior to change of threshold followed by linear deterioration thereafter. R^2 ^= 0.62, *P* < 10^−4^.

Six patients became symptomatic at a mean age of 49.6 years (range 42–58) while under review in the Cohort study; diagnosis was confirmed at an average 11.8 months later, and after a further average 5.7 months, the symptoms become significant in that they affected activities of daily living. The last examination before becoming symptomatic had been normal in five patients; the other, who had a parietal meningioma removed some years before, had an extensor plantar response and minor loss of vibration appreciation in the leg. The six patients were examined 3 to 6 months after first developing symptoms all of whom complained of feeling unsteady and three of lower limb dysaesthesiae. Examination at that time is summarized in Fig. 1D. No patients had abnormal neurological signs in the upper limbs at the time they first developed symptoms. The proportion of patients exhibiting these abnormal signs increased after conversion (Fig. 1D). Two patients died 49 and 72 months after becoming symptomatic during which time they developed pyramidal weakness greater in the lower limbs, increasing ataxia, sensory loss, incontinence and cognitive decline ultimately becoming akinetic and mute.

#### Patients who were asymptomatic throughout study

Of the 11 patients in this group one had absent lower limb reflexes. No neurological signs were elicited in the other 10 patients.

### Imaging

Serial MRIs of the brain were obtained in 11 patients who were symptomatic at some time during the study, one of whom also had an MRI of the spine; two to five studies were performed in all of these patients. Signal abnormality in the cerebral cortex was detected in two patients and in the basal ganglia in one patient, 6 and 7 months after developing symptoms. Cortical and cerebellar atrophy was detected in six. In all cases these changes were observed after symptom onset. The one spine MRI was normal.

### Neurophysiological results

All studies in the upper limbs were normal. In the lower limbs sensory and motor conduction, F wave, histamine flare and threshold tracking were normal in all patients in whom the test was done.

### Abnormalities in patients who were symptomatic on entry to the study

The H reflex was normal in three of five patients when first assessed at 7–17 months after symptom onset and was abnormal in all by 41 months. Similarly, thermal thresholds were normal or borderline in three patients initially but were abnormal in all 10–41 months after first developing symptoms (Fig. 1). Histamine-induced itch, which was not consistently assessed, was abnormal in all patients 8–41 months after symptom onset.

### Findings in patients who became symptomatic

Six patients who were asymptomatic when first seen developed symptoms of the disease during follow-up. Symptom onset was defined as the time at which any symptom developed that subsequently progressed and was retrospectively thought to be part of the Gerstmann-Sträussler-Scheinker syndrome. These six patients developed significant symptoms affecting normal daily activities (disability onset) between 3 and 30 months after symptom onset [mean = 17.5 months, standard deviation (SD) = 9.6] and were confirmed by their consultant as having developed the disease between 3 and 18 months after symptom onset (mean = 11.8, SD = 7.2). Changes in neurophysiological parameters are shown relative to the onset of significant symptoms in the charts (Fig. 1).

The H reflexes were normal in all patients prior to symptom onset and were lost in four patients 3–14 months later, in all four cases prior to or at the same time as clinical diagnosis and/or the development of disability; in the other patients the H reflex was obtained when last measured 4 and 30 months after conversion (Fig. 1C).

Threshold for detection of hot was normal (<44°C), except on one occasion, in all patients before symptom onset. Cold threshold was below 23°C in one patient 6 months before developing symptoms and normal in the rest. When seen 3–14 months after symptom onset, and prior to onset of significant symptoms, or clinical diagnosis, all patients had abnormality of thermal sensation; in four both hot and cold thresholds were abnormal, and in the other two only one modality was abnormal (Fig. 1A and B). Average hot threshold was 37.7°C in the last examination prior to symptom onset, and 45.0°C in the following examination prior to clinical diagnosis and disability onset (*n = *6, SD = 1.43, paired *t*-test versus previous examination, *P* = 0.0012). A striking feature was the change in threshold after symptom onset; in the case of hot threshold the change was of the order of 5–8°C and cold detection was lost in all but one case within 30 months.

When thermal threshold changes from baseline were examined in patients who converted using a range of linear and non-linear statistical models, the best fit (R^2 ^= 0.62) was observed with a segmented linear model. This model assumed no change in thermal thresholds (slope = 0) until 30 months prior to symptom onset, followed by linear deterioration (Pearson correlation *P* < 0.0001, 2.9°C/year, 95% 1.8–4.0°C/year; Fig. 1E).

Itch was not consistently determined. However, three patients had lost this sensation when assessed at 22–27 months after symptom onset and two still reported itch when last assessed at 15 and 16 months after symptom onset. One patient with the shortest follow-up period following conversion was not tested but had experienced this sensation on histamine injection before. The histamine flare was normal in all.

### Findings in persistently asymptomatic subjects

There were neurophysiological abnormalities in three asymptomatic patients aged 42–48 years. Two had an absent H reflex in the lower limbs and two had abnormal thermal detection thresholds including one of those with abnormal H reflexes. Two of these patients did not detect an itching sensation with histamine injection. Serial measurements of thermal thresholds over a period of up to 61 months showed no consistent change with small variation between measurements greater for hot (9%) than cold (<5%). Similarly, in those patients with an H reflex there was a small variation between measurements of <10% but no trend. Average cold threshold was 28.2°C (*n = *19 assessments, 11 patients, SD = 2.2), average hot threshold was 37.0°C (SD = 3.7) and H latency was 26.9 ms (SD = 7.3) (Fig. 1).

### Neuropathological results

Abnormal prion protein deposits were detected with both 12F10 and ICSM35 (data not shown) antibodies. Generally 12F10 showed more prominent labelling, resulting in a more widespread and dense abnormal prion protein staining and importantly, also in a wider range of labelling patterns. There was variability in the labelling intensity between the cases, in that an increase of the post mortem formalin fixation interval attenuated immunostaining intensity.

#### PrP deposits in the sensory pathway

**Figure 2:**
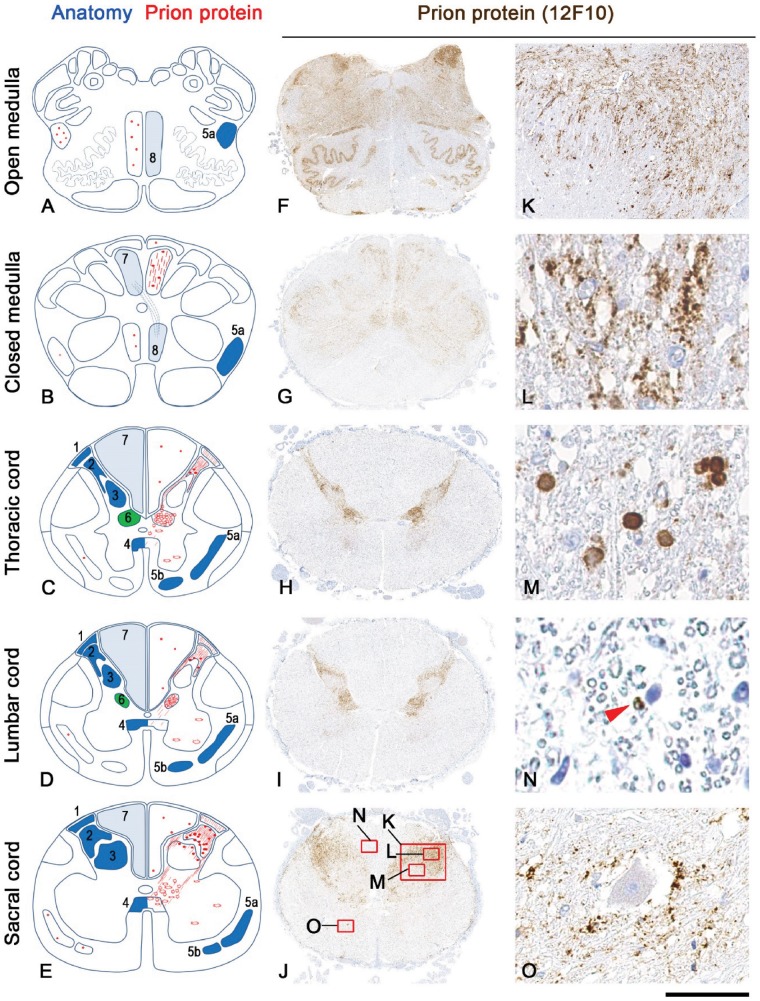
**Prion protein pathology in the spinal cord and brainstem of a representative patient with a *PRNP* P102L mutation.** (**A**–**E**) Schematic demonstration of the extent and distribution of the prion protein pathology in the spinothalamic pathway (dark blue: 1 Lissauer’s tract, 2 substantia gelatinosa Rolandi, 3 nucleus propius, 4 spinothalamic decussation, 5a lateral spinothalamic tract, 5b anterior spinothalamic tract), Clarke’s (dorsal) column (green 6) and posterior column-medial lemniscus pathway (light blue; 7 posterior columns, 8 medial lemniscus). (**F**–**O**) Immunostaining for the abnormal prion protein with 12F10 antibody: Overview of the abnormal prion protein deposits within the spinal cord and brainstem (**F**–**J**). In the brainstem (**F**–**G**) the prion pathology is seen with widespread distribution and is not restricted to the sensory pathways. At all levels of the spinothalamic pathway there is dense synaptic labelling (**K**–**L**) in the Lissauer’s tract, marginal nucleus of the spinal cord (lamina 1), substantia gelatinosa (lamina 2), nucleus proprius (lamina 3/4), lamina 5 and 6, central white commissure and Clarke’s (dorsal) nucleus. In the substantia gelatinosa in addition to synaptic labelling there are occasional small plaques (**M**). In the white matter tracts of the spinothalamic and posterior column-medial lemniscus pathway there are occasional circular intra-myelin inclusions (**N**, arrowhead). In the anterior horns there is occasional peri-neuronal labelling around the large motor neurons and small interneurons (**O**). Scale bar = 50 µm in **K**–**O**.

In the posterior nerve roots, in the immediate vicinity to the spinal cord, occasional labelling in the unmyelinated and pauci-myelinated areas was evident at the sacral level. In the spinothalamic pathways, dense labelling was seen in the Lissauer’s tract and marginal nucleus of the spinal cord (lamina 1), substantia gelatinosa (lamina 2, termination of C fibres), nucleus proprius (lamina 3 and 4 , major termination of Aδ fibres), dorsal nucleus in thoracic and high lumbar cord (lamina 5 and 6), and central white commissure (lamina 10) comprising crossing fibres of the spinothalamic pathway (Fig. 2). In the white matter tracts occasional ring-like deposits were detected along the whole length of the spinothalamic pathway.

**Figure 3:**
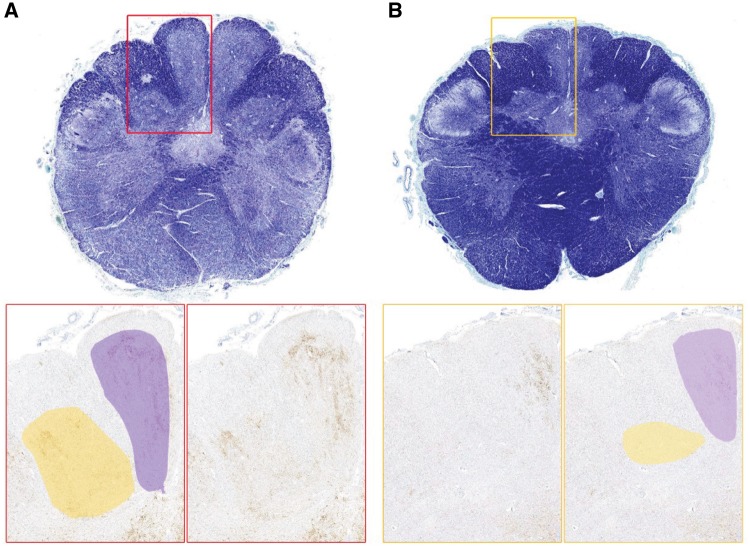
**Prion protein pathology in the gracile and cuneate nuclei. **In four patients there are widespread abnormal prion protein deposits in both nuclei with no difference in the labelling intensity and coverage when assessed quantitatively in three patients (**A**, shown one representative case). In one patient (**B**) quantitative assessment confirms that the gracile nucleus (purple in cartoon) is more affected than the cuneate nucleus (yellow in cartoon). Luxol fast blue/cresyl violet stain above, 21F10 antibody stain below.

The posterior column-medial lemniscus pathway showed only rare deposits in the white matter tracts, while the gracile and the cuneate nuclei showed dense abnormal PrP deposits (Fig. 3). These results in two patients were confirmed using a quantitative histological imaging system (Definiens Developer). In one patient the gracile nucleus was more affected than the cuneate whilst no difference in the labelling intensity and coverage on quantitative assessment was observed in the other patient (Fig. 3B). In Clarke’s (dorsal) nucleus (lamina 5 and 6) labelling was prominent (Fig. 2H and I) and peri-neuronal labelling was seen around the large motor neurones and small interneurons within the anterior horns (Fig. 2O). The thalamus contained frequent synaptic deposits and plaques of various sizes.

In the neocortical areas, including parietal cortex, prion pathology was widespread and similar to that reported previously (Webb *et al.*, 2008). No definite prion pathology was seen in the anterior nerve roots of the cauda equina or in the peripheral nerves (median, sural and ulnar nerves in two patients, sciatic in one and radial in another patient).

## Discussion

There is a reasonable expectation that treatments for neurodegenerative diseases may be ineffective unless given in the earliest stages of disease before substantial neuronal damage has occurred. This motivation has led to increased interest in monogenetic forms of dementia in which individuals at high-risk of disease can be identified, studied and potentially treated prior to or soon after symptom onset (Tabrizi *et al.*, 2009). To achieve this, it is necessary to develop biomarkers that are abnormal in symptomatic patients carrying the relevant mutation then to follow a sufficiently large cohort of patients with the mutation from the asymptomatic to the symptomatic phase to determine when the biomarker becomes abnormal and therefore determine if it predicts disease milestones. In this paper we have shown that neurophysiological abnormalities predate the clinical diagnosis and disability in inherited prion diseases due to a mutation in the prion protein gene (P102L) in which ataxia of gait, lower limb areflexia and lower limb sensory changes are the usual initial findings; further, these measures may be of value in monitoring disease progression. We also describe the pathological basis for these clinical and neuro-physiological findings in a subset of patients.

The first objective in this study was to determine if neurophysiological testing of thermal sensation and H reflex, being more objective and quantifiable than the usual clinical examination, is of value in predicting clinical milestones in Gerstmann-Sträussler-Scheinker disease due to P102L. The progression of Gerstmann-Sträussler-Scheinker disease passes from asymptomatic through clinical stages, with initial symptoms often being non-specific (tingling in the feet, alteration in mood, personality or gait), and only later are the symptoms and signs clear enough for clinical diagnosis, and/or severe enough to impair activities of daily living. Neurophysiological measurement of thermal threshold and the H reflex did not predict the earliest milestone of symptom onset in individual cases, but became abnormal shortly thereafter, and predated clinical diagnosis and the development of disability. Regression analyses of groups of patients converting to clinical disease suggested that the warm detection threshold in the legs starts to change ∼3 years before significant (disabling) symptoms are present. The most immediate implication of our observations is for a proximity biomarker in Gerstmann-Sträussler-Scheinker disease: a new abnormality of the hot or cold lower limb thermal threshold or the H reflex predicts clinical diagnosis and could support early recruitment to clinical trials through eligibility criteria.

In prion diseases, a special complexity is that age at clinical presentation may extend across an adult lifespan, and the clinical features at presentation are heterogeneous between and within families (Mead, 2006). This means that estimates of age at clinical onset are highly unreliable (standard deviation of age of clinical onset typically ∼10 years for inherited prion diseases). A limitation of our work is the relatively small number of participants who converted to clinical disease during the study. On the other hand, an advantage of our study is the fact that clinical milestones were observed and not estimated. Large multinational studies of monogenetic causes of dementia e.g. DIAN and GENFI, use predictions of clinical onset to allow inference of biomarker change prior to symptom onset (Bateman *et al.*, 2012; Rohrer *et al.*, 2015; Ryan *et al.*, 2016). Differences between estimated and measured clinical onset may lead to an overestimate of how early biomarker change occurs. It is notable that the biomarker changes reported in this study are much closer to clinical onset than was estimated in these related neurodegenerative disorders.

An additional objective was to see if there was increase in the magnitude and number of abnormalities on neurophysiological testing once the patient was symptomatic. Unfortunately the H reflex, which is dependent on the Aα afferent nerve fibres, was suddenly lost with no evidence of a gradual increase in latency. This bimodal distribution is explained by the narrow range of velocities in these Aα fibres which results in the sudden loss of the response rather than a gradual change. An attempt was made to overcome this limitation by calculating the ratio of the amplitude of the H reflex to the primary muscle response (M) but this was extremely variable within a subject and was not reliable. In contrast thermal thresholds, although dependent on the subject reporting the onset of sensation, gives an absolute measurement of thresholds. In the present study, in spite of the limited number of patients studied, hot thermal thresholds did sequentially decline in all and might be useful as an objective measure of change in clinical trials. Thermal thresholds might be included as a secondary endpoint in clinical trials, testing for a change in slope of deterioration over time compared with controls. We have not studied enough patients to estimate the potential power of such a biomarker. The H reflex would be less attractive because of its binary outcome. In conclusion, the present study provides proof of principle that neurophysiological assessments (particularly the hot thermal threshold) might be of value in monitoring transition and progression of Gerstmann-Sträussler-Scheinker disease due to P102L mutation but a larger cohort of patients, with a prospectively defined biomarker will be required to determine the confidence intervals of the different parameters studied.

A second objective was to delineate the anatomical basis for the symptoms and investigation findings in this study. The present study showed no evidence of abnormal peripheral nerve dysfunction. The loss of the H reflex is consistent with damage proximal to the dorsal root ganglion or within the lumbar spinal cord involving the Aα afferent pathway to the anterior horn cells; an earlier study came to the same conclusion (Salsano *et al.*, 2011). The abnormal thermal detection thresholds indicate an abnormality in the spinothalamic pathways between the periphery and the cerebral cortex; peripheral cold detection is dependent on Aδ fibres while hot detection is mediated by C fibres (Fowler *et al.*, 1988). The abnormality detected in the lower limbs were unlikely to be due to involvement of the peripheral pathway as the histamine flare response, dependent on non-mechanoreceptor C fibres, was intact in those patients who did not report itching to histamine injection, a sensation also dependent on the same group of fibres (Aronin *et al.*, 1987). This indicates damage to the spinothalamic system within the CNS between the substantia gelatinosa and thalamus. Finally, the abnormality of vibration sense appreciation on clinical examination implies damage to the Aα afferents passing through the posterior columns to the posterior column nuclei.

The neuropathological findings largely confirmed these predictions. Dense synaptic deposits of abnormal prion protein were identified in the posterior horns of the lumbosacral spinal cord, in keeping with a previous case report (Yamada *et al.*, 1999). However, in our study PrP^Sc^ deposition was more widespread; deposits were identified in Clarke’s column (nucleus dorsalis), and the posterior column nuclei, with some deposition in the spinothalamic tracts and dorsal columns. In addition there was peri-neuronal labelling around the large motor neurons and small interneurons within the anterior horns. Interestingly, prion protein deposits were present not only in the lumbosacral cord but in the same areas also in the thoracic and upper cervical cord but these changes probably reflect a late development given the normal neurophysiology in the upper limbs in the early stages of the disease.

These neuropathological findings in the lumbosacral cord are a sufficient explanation of the neurophysiological abnormalities in the lower limbs. The prion protein deposition in the substantia gelatinosa, spinothalamic tracts and, to a lesser extent, in the small myelinated (Aδ) fibres in the lateral part of the proximal dorsal roots explains the abnormal thermal thresholds and loss of itch sensation, while the abnormality in the posterior column nuclei explains the impaired vibration sensation. The H reflex abnormality could reflect either impaired conduction in the Aα fibres passing through the affected area of the neuropil in the dorsal horn or involvement of the motor neurones. Similarly, the late development of leg weakness could be due to anterior horn cell loss in the lumbar region. The early development of ataxia of gait has been traditionally explained by cerebellar involvement in Gerstmann-Sträussler-Scheinker disease, but the extensive deposition of PrP^Sc^ in Clarke’s column where collaterals of the Aα fibres terminate, the cells of which are not found in the cervical cord, could well be the proximate cause of the unsteadiness; such an explanation would also account for the relatively preserved limb coordination during the initial course of the disease.

12F10 antibody revealed more extensive pathology when compared with findings seen using ICSM35 antibody. Less prominent, albeit not complete absence of labelling of the abnormal prion protein using ICSM35 antibody, can be explained by previously shown inability of ICSM35 to recognize human abnormal prion protein with lysine at position 102 and the documented spectrum of PrP wild-type involvement with subsequent conformation to PrP^Sc ^in the disease process (Wadsworth *et al.*, 2006). Prolonged post-mortem interval, long formalin fixation time and the selection of the antibodies are plausible explanations for completely negative labelling for the abnormal prion protein in the spinal cord in two Gerstmann-Sträussler-Scheinker cases described in another study (Lee *et al.*, 2005).

## Conclusion

This study shows that the initial symptoms and signs in most cases of Gerstmann-Sträussler-Scheinker disease are likely to be due to abnormal prion protein deposition within the lumbosacral cord and the posterior column nuclei. Neurophysiological assessment did not predict the onset of the initial symptoms of the disease but predated significantly disabling symptoms and clinical diagnosis, and subsequently progressively deteriorated, therefore may be of value as an early biomarker.
