# Early neurophysiological biomarkers and spinal cord pathology in inherited prion disease

**DOI:** 10.1093/brain/awz040

**Published:** 2019-02-18

Peter Rudge, Zane Jaunmuktane, Harpreet Hyare, Matthew Ellis, Martin Koltzenburg, John Collinge, Sebastian Brandner, Simon Mead. Early neurophysiological biomarkers and spinal cord pathology in inherited prion disease. *Brain*, awy358, https://doi.org/10.1093/brain/awy358.

The authors would like to apologise for two of the authors' affiliations being listed incorrectly in the original manuscript, they are listed correctly below:

John Collinge^1,2^ and Simon Mead^1,2^

1 National Prion Clinic, National Hospital for Neurology and Neurosurgery, University College London Hospitals NHS Foundation Trust (UCLH), London, UK 2 MRC Prion Unit at UCL, Institute of Prion Diseases, 33 Cleveland St. London, W1W 7FF, UK

The manuscript has been corrected online.

